# Modelling and Predicting Population‐Level Growth With Individual‐Level Information

**DOI:** 10.1002/sim.70421

**Published:** 2026-02-22

**Authors:** Tuuli Kauppala, Tuomo Susi, Sangita Kulathinal

**Affiliations:** ^1^ Department of Data and Analytics Finnish Institute for Health and Welfare Helsinki Finland; ^2^ Department of Mathematics and Statistics University of Helsinki Helsinki Finland

**Keywords:** Bayesian prediction, bivariate hierarchical model, growth modeling, growth prediction, hierarchical linear model

## Abstract

The development of height, weight, and body mass index (BMI) in children has been the subject of considerable interest due to secular changes in growth patterns, such as increases in height and rising obesity rates. Predicting growth in a target population is particularly challenging when the population comprises of individuals with and without past growth data. In this study, we present three approaches for the joint prediction of height and weight in that situation. The predictive performance of each approach is evaluated using a range of measures that assess different properties of the prediction distributions. We also compare the approaches to interpret their clinical relevance, particularly in terms of prediction accuracy. The developed prediction approaches vary in their use of past growth data. We predict growth for a target population of children aged 4–11 years in 2021, residing in three municipalities in Finland. We employ longitudinal register data on height and weight, collected from children aged 2–11 years between 2014 and 2020 in these municipalities to construct a Bayesian hierarchical linear model (HLM) for growth prediction. Additionally, we estimate posterior unconditional distributions of height, weight, and BMI for within‐sample model validation. The inclusion of individual‐level data in the predictions reduced the divergence from observed measurements, particularly for weight and BMI. This is important given the skewed distribution of the measurements with increasing age. Incorporating individual‐level information is also beneficial for child‐specific predictions. Our study highlights the importance of multiple prediction checks to understand the flaws and strengths of each prediction approach.

**Abbreviation**
HLM: hierarchical linear model

## 1 Introduction

Childhood growth is a widely studied topic with a diverse range of models and study designs used to describe it, particularly in terms of growth charts, individual development and heritability [1, 2]. Predicting growth for current and future generations is a timely issue, especially in light of the rising rates of childhood obesity reported in multiple countries [3], making it an important focus from a public health perspective. Most studies on growth prediction methodology focus on forecasting individual height development. At the population level, long‐term Body Mass Index (BMI) forecasts using simulated longitudinal datasets based on cross‐sectional data have been introduced [4, 5].

The primary focus of this study is on population‐level growth prediction for a target population that includes both individuals with and without repeated measurements of height and weight. Such data are typical of high‐coverage register datasets spanning multiple years, which are expected to become more common, as noted by Oravesz et al. [6] and Sund et al. [7]. However, it remains unclear whether prediction accuracy could be improved by maximizing the use of individual‐level information. Our interest is to explore this question using appropriate methodology for prediction evaluation, motivated by a dataset in which approximately 80% of the target population has individual‐level growth data. In this study, growth is defined as the age‐related development of height, weight, or BMI (${\text{kg/m}}^{2}$), where BMI is the ratio of weight (kg) to the square of height ($m^{2}$).

In Finland and globally, the relationship between height and weight is complex as the population has not only become heavier but also taller [8, 9]. Prediction of the BMI may look attractive if the goal is to understand the development of childhood weight along height, but that approach is inadequate when both height and weight of the population change. For example, a BMI value of $18.5\;\text{kg}/m^{2}$ corresponds to multiple combinations of weight and height, for example, (40, 147) and (45, 156). Attempts to model height and weight jointly have been made, for example, in a study by Deshpande et al. in adult women [10]. This approach also enables bivariate prediction. Moreover, bivariate modelling and prediction of height and weight have been identified as important areas of future research in growth studies [1, 11].

In Kauppala [12], a Bayesian bivariate hierarchical linear model (HLM) was developed for estimating growth parameters in a longitudinal setting. This model was chosen to address the imbalanced nature of the data and to provide estimators and predictions for both population and individual growth. An advantage of Bayesian HLMs is their ability to account for uncertainty on many levels, and to provide posterior predictive distributions. In this work, we study the empirical properties of three different prediction approaches based on that model architecture. The use of Bayesian bivariate HLMs in longitudinal settings is currently rare, with only a limited number of studies to reference. For example, Carles et al. examined and evaluated a bivariate Bayesian postnatal growth model using both simulation data and a real‐world example [13]. Other studies have proposed Bayesian longitudinal bivariate models for investigating growth, including for within‐person changes [6], for coral growth [14], for embryonic growth [15], and for changes in individual learning [16].

The main goals of the study are:

- Prediction of height, weight and BMI for a target population with and without available previous growth information. This includes the development of prediction approaches that vary in the handling of past growth information. To enable evaluation of the predictions, we defined a target population whose current height and weight data are available in Finnish nationwide health registers.
- Comparison of the prediction approaches in terms of population‐level prediction accuracy. We aim for using multiple evaluation approaches to understand the strengths of each prediction method. Diverse measures that emphasize different properties of the prediction outcomes are applied.
- Interpretation of the results according to clinically meaningful prediction accuracy.
- Estimation of posterior unconditional distributions of height, weight and BMI for within‐sample model checks.

A secondary goal is to evaluate prediction accuracy for individual children, for example, when the height and weight observations are not treated as interchangeable.

In Section 2, we describe the longitudinal health register‐based dataset used in this study. We define different approaches and evaluation methods for prediction in Section 3, and describe the results in Section 4. A point‐by‐point discussion of various aspects of prediction is presented in Section 5.

## 2 Dataset

The dataset used in this study is derived from the Finnish Primary Health Care Visits register (Avohilmo) [17, 18]. The usage and maintenance of the collected and processed dataset is executed by the FinLapset project at the Finnish Institute for Health and Welfare (THL) [19]. For the purpose of this study, the time unit of interest in growth is one year, and we selected one representative measurement per year for each individual.^1^ If multiple measurements per year were available, the one closest to the birthday was chosen for the analysis.

We limited the exploration and modelling to repeated height and weight measurements of male children from the calendar period 2014–2020 from three municipalities of southern Finland (Uusimaa region), namely Vihti, Kirkkonummi, and Tuusula. The population whose measurements were used to learn the model's parameters consists of 11 532 children aged 2–11 years between 2014 and 2020 (Contributing Group). At least two measurements for different age years were required. For prediction purposes and out of sample validation, we defined a target population consisting of male children measured in the age group of 4–11 in 2021 residing in the above‐mentioned municipalities (Target Group). The Target Group consists of 3535 children, some of whom are also part of the Contributing Group. To evaluate the predictions in more detail on the population‐ and individual‐level, we defined the intersection of the Target Group and Contributing Group (Contributing Group‐2021), which consists of individuals with previously available growth information and measurements in 2021.

**FIGURE 1:**
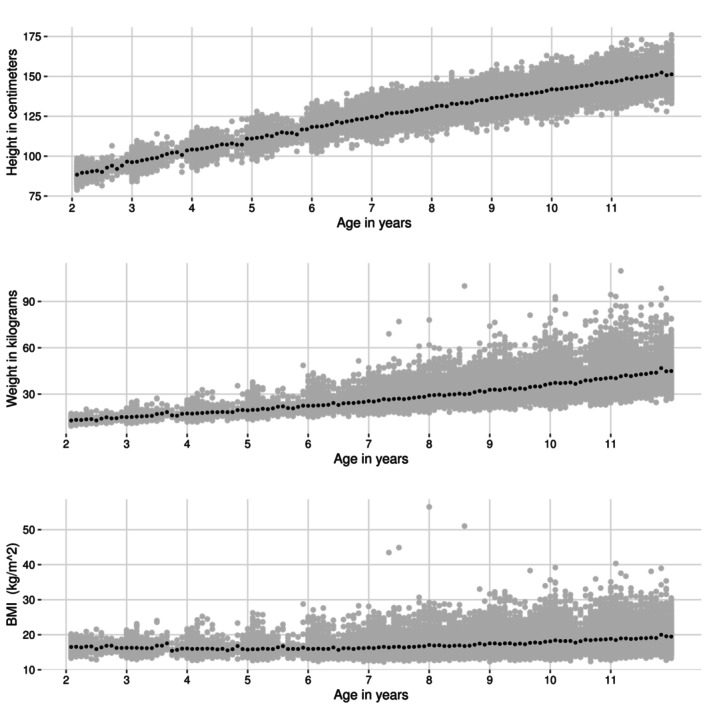
Height, weight and BMI distribution of the Contributing Group (defined in Section 2). The black dots indicate means for each age month.

Table 1 presents the Target Group size by age and contribution status. Of the 3535 individuals in the Target Group, 766 (≈2%) were not part of the Contributing Group in 2021. The distribution of observations was relatively uniform across ages. Figure 1 illustrates the distributions of height, weight, and BMI by age in the Contributing Group. We observe that height (rounded to the nearest cm) and weight are measured closer to the individual's birthdate during the preschool years (ages 2–6), after which measurements are more evenly distributed across age. Height is symmetrically distributed for all age groups, while the weight displays skewness and deviation from the mean with age. A similar trend is observed for BMI.^2^

**TABLE 1:** The sizes of target group, consisting of contributing group‐2021 and its complement group denoted as non‐contributing group‐2021.

	Target group	Contributing group‐2021			Non‐contributing group‐2021
Age in 2021	N (total)	N (in Model 2)	N (Model 1)	N (Model 1 and 2)	N (in neither models)
4	455	0	174	0	281
5	437	0	337	0	100
6	536	0	444	0	92
7	445	0	339	0	106
8	406	38	145	160	63
9	390	60	61	215	54
10	387	141	16	186	44
11	479	299	1	153	26
Total	3535	538	1517	714	766

*Note:* Model 1 denotes model for 2–5 year olds in 2014–2020 and Model 2 the model for 6–11 year olds in 2014–2020.

## 3 Methods

We fitted bivariate HLMs separately for age groups 2–5 and 6–11 years. For brevity, we denote models for age groups 2–5 and 6–11 as Model 1 and Model 2, respectively. We transformed the weight observations to reduce the skewness of their distribution and make it bell‐shaped for every age year. Height observations were log‐transformed to define log (BMI) as a difference between log(weight) and log(height). We examined the transformation effect with Q‐Q plots stratified by age year. The log‐transformation reduced the skewness of the weight distribution without compromising the normality of the height distribution across all age groups.

### 3.1 Model Description

Let the response variable be a matrix $Y=((y_{\mathrm{ti}}))$ of dimension $\left((\sum_{i=1}^{n}T_{i})\times 2\right)$, where $T_{i}$ represents the number of measurements of the two variables of interest for individual i, and n equals the number of individuals. In our case, we define $y_{\mathrm{ti}}=(y_{H_{ti}},y_{W_{ti}})$, where the first element is the log(height), and the second is the log(weight) measured at time $t_{i}=1,2,\ldots T_{i}$ for an individual i. We denote the model parameters $\eta_{i}$ as $\left(\eta_{0i},\eta_{1i}\right)$, where $\eta_{0i}$, $\eta_{1i}$ represent height and weight parameters, respectively, of individual i. The population‐level parameter β stands for $\left(\beta_{H0},\beta_{W0},\beta_{H1},\beta_{W1}\right)$. We use μ and σ with the appropriate subscript to denote a mean vector and a diagonal standard deviation matrix, and the subscript gives the corresponding variable. Random variables are denoted in upper case and corresponding observations in lower case.

We assume that the outcomes are independent and normally distributed given individual level random effects $\eta_{i}$. Furthermore, we assume that $\eta_{i}$ themselves have bivariate normal distribution capturing possible dependencies between the two response variables. The full Bayesian hierarchical model is:

(1) $$\begin{array}{ll} \left(Y_{\mathrm{ti}}|a_{ti},\eta_{i},\sigma_{e}^{2}\right) & ∼N(\eta_{0i}+\eta_{1i}a_{ti},\text{diag}(\sigma_{e_{H}}^{2},\sigma_{e_{W}}^{2})) \end{array}$$

(2) $$\begin{array}{ll} \left(\eta_{i}|\beta ,\Omega \right) & ∼N(\beta ,\Omega )\\ \sigma_{e_{H}}\;\text{and}\;\sigma_{e_{W}} & ∼\text{inv‐Gamma}(0.5,0.5)\\ \beta_{H0},\;\beta_{W0},\;\beta_{H1},\;\beta_{W1} & ∼N(a,b). \end{array}$$

At the first hierarchy level (Equation (1)), $a_{ti}$ is the age of individual i at time t, and $\sigma_{e_{H}}$ and $\sigma_{e_{W}}$ are standard deviations within individual's height and weight, respectively. Note that the standard deviations are assumed to be the same for all t, and are independent and distributed according to an inverse‐gamma distribution. At the second hierarchy level (Equation (2)), parameters β are interpreted as the population‐level expectations for the individual‐level intercept: $\beta =(E(\eta_{0i}),E(\eta_{1i}))$, where $E(\eta_{0i})=(\beta_{H0},\beta_{W0})$ and $E(\eta_{1i})=(\beta_{H1},\beta_{W1})$. The covariance matrix Ω shows between‐person variation in log(height) and log(weight), and the correlation between these traits.

The prior choices are mostly uninformative. In case of parameters β, informative priors are chosen based on estimates of the corresponding parameters of the frequentist linear model. In Kauppala (2021) (pages 33, 41, and 70), we show that the posterior predictive distribution's characteristics for hyperparameters β are not sensitive to these informative prior choices [12], as both the parameter estimates and their standard errors remained unchanged up to the third decimal place. The hyperprior distribution for the covariance matrix Ω is specified using scale and correlation components τ and λ as follows:

Ω=diag matrix(τ)λdiag matrix(τ),

which is the recommended prior choice for the covariance matrix in the Stan user manual (section “Multivariate priors for hierarchical models”) [20]. The priors for τ follow a half‐Cauchy distribution with a scale of 2.5. For the parameter λ we use Lewandowski‐Kurowicka‐Joe correlation prior,^3^ as described in detail in Kauppala [12, pp. 24‐25].

### 3.2 Bayesian Computation

The statistical computation was performed using the R interface to the STAN software [20, 22, 23]. Due to the known computational and convergence challenges of HLMs, we performed multiple convergence checks. We inspected the traceplots of the parameters β, $\sigma_{e}$, τ, λ and ensured that no divergent transitions were reported by Stan software.^4^ Efficient sample size (ESS) values and Rubin‐Gelman R‐hat estimates were examined and their values are reported in Supporting Information C [24]. The inspection of those is the recommended procedure for convergence checks when using iterative simulation draws in statistical inference (see Gelman et al. [21, pp. 284–287]). The code for the Stan model is available in the Supporting Information E [24].

### 3.3 Unconditional Distributions of Height, Weight and BMI

Our interest is also in the distribution of the random vector $Y_{ti}$ (log height, weight, or here, BMI) and $X_{ti}=\exp(Y_{ti})$, after integrating out the individual‐level random effects. Deriving the unconditional distribution parameters for a conditional random variable is useful because it provides us with parameters for the whole population with straightforward interpretation, for example for within‐sample checks.

Under the hierarchical model specified by Equations (1) and (2), the distribution of $Y_{ti}$ is bivariate normal, and $X_{ti}$ has a bivariate log‐normal distribution. Below, we derive the parameters for these distributions.

The notation is the same as in Section (3.1), including the equality $Y_{ti}=log(X_{ti})$. Similarly, $\sigma_{e}^{2}$ will denote $\text{diag}(\sigma_{He}^{2},\sigma_{We}^{2})$, and the same will apply for level 2 (Equation (2)) variance and covariance parameters. The expectations and variances of $Y_{ti}=(Y_{H_{ti}},Y_{W_{ti}})$ for given $a_{t}$ and $\left(\beta ,\sigma_{e}^{2},\Omega \right)$, averaging over $\left(\eta_{0i},\eta_{1i}\right)$, are derived as follows.

$$\begin{array}{ll} E(Y_{ti}) & =E(E(Y_{ti}|\eta_{0i},\eta_{1i},a_{ti}))=\beta_{0}+\beta_{1}a_{t},\\ \mathrm{Var}(Y_{ti}) & =E(\mathrm{Var}(Y_{ti}|\eta_{0i},\eta_{1i},a_{ti}))+\mathrm{Var}(E(Y_{ti}|\eta_{0i},\eta_{1i},a_{ti}))\\ & =\sigma_{e}^{2}+\mathrm{Var}(U\eta_{i})=\sigma_{e}^{2}+U\Omega U^{t}, \end{array}$$

where $U=\left(\begin{array}{llll} 1 & 0 & a_{t} & 0\\ 0 & 1 & 0 & a_{t} \end{array}\right)$ and $U\eta_{i}$ gives the expectation in Equation (1).

The expectation and variance of $Y_{{BMI}_{ti}}=l^{t}Y_{ti}$, where $l=\left(\begin{array}{l} 1\\ -\;2 \end{array}\right)$ and height is in meters, can now be easily derived as

$$E(Y_{{BMI}_{ti}})=l^{t}E(Y_{ti})=l^{t}(\beta_{0}+\beta_{1}a_{t}),$$

and

$$Var(Y_{{BMI}_{ti}})=l^{t}\mathrm{Var}(Y_{ti})l=l^{t}(\sigma_{e}^{2}+U\Omega U^{t})l.$$

The unconditional distribution of X can be obtained as stated in Supporting Information A [24].

### 3.4 Prediction Approaches

We divided prediction into three types:

1. Prediction based on level 1 (individual) parameters from Equation (1) (Pred‐I).
2. Prediction based on level 2 (population) parameters from Equation (2) (Pred‐P).
3. Prediction approach combining Pred‐I with Pred‐P depending on availability of individual parameters (Pred‐C),

In Bayesian framework, one typically provides the posterior predictive distribution for any unobserved trait $\tilde{y}$. This distribution is the conditional distribution of $\tilde{y}$ given the data and is defined as:

(3) $$\begin{array}{l} p(\tilde{y}|y)=\int p(\tilde{y}|\theta )p(\theta |y)d\theta , \end{array}$$

where θ are unknowns of the probability model (both the data and the prior models). For brevity, we will later refer to expectation of the child i's posterior predictive distribution $\tilde{y_{i}}$ for age $a_{t}$ equivalently as prediction of $\tilde{y_{i}}$. A practical description of prediction for HLM's is given in Gelman et al. [25, pp. 272‐273]. Our notations below follow the description.

The prediction problem for an individual with past growth information, known as the conditional (or partial) prediction problem, is discussed by Fearn in the context of Bayesian longitudinal growth HLMs, alongside the prediction problem for a new individual [26]. The solutions are based on parametric methods focused on individual‐level prediction. This study emphasizes the use of individual‐level information for population‐level prediction.

#### 3.4.1 Individual Parameter‐Based Prediction (Pred‐I)

In our case, the Pred‐I prediction approach refers to using level 1 information of the growth model (Equation (1)). The Pred‐I approach can only be used if the individual in the Target Group is in the Contributing Group‐2021, as otherwise there are no individual parameters available for prediction (Table 1). The Pred‐I approach is described below.

1. Prediction for an individual aged 4–5 years in 2021.
  ∘ We use the posterior distributions of the individual‐level intercept and slope from Model 1 (age 2–5) in the prediction (see below).
2. Prediction for an individual aged 6–11 years in 2021. Here we have three possibilities.
  ∘ In case of the individual having observations when aged 2–5 years but none when aged 6–11 years (and therefore contributing to Model 1 only), we use only the height and weight prediction for age of six from Model 1 and treat it as the individual‐level intercept.
  ∘ In case of the individual having observations when aged 6–11 years but none when aged 2–5 years (and therefore contributing to Model 2 only), we use the individual‐level intercept and slope posterior distribution estimates directly from Model 2.
  ∘ In case of the individual having some observations when aged 2–5 years as well as 6–11 years (and therefore contributing to Models 1 and 2), we use the level 1 intercept and slope posterior distribution estimates from Model 2.

The individual child's growth parameters $\eta_{i}$ are used based on the posterior distribution

$$(\eta_{i}|\beta ,\Omega ):(\eta_{i}^{\left(1\right)},\eta_{i}^{\left(2\right)},\ldots ,\eta_{i}^{\left(m\right)}),$$

j=1,…,m being the sample size of the posterior distribution and i=1,…n the child's index. We use the MCMC parameter sets evaluated for the individual child i with a total of m=1000 samples per child. In this way, uncertainty in the estimation of the parameter $\eta_{i}$ is considered.

For each $\eta_{i}^{\left(j\right)}$ we sample a posterior distribution $p(y_{i}^{\left(j\right)}|\eta_{i}^{\left(j\right)})$ from Equation (1) of the HLM:

$$y_{i}^{\left(j\right)}|\eta_{i}^{\left(j\right)},\sigma_{e}∼N(\eta_{i1}^{\left(j\right)}+\eta_{i2}^{\left(j\right)}a_{t},\sigma_{e}),$$

resulting in a sample $\left(y_{i}^{\left(j)(1\right)},y_{i}^{\left(j)(2\right)},\ldots ,y_{i}^{\left(j)(k\right)}\right)$, for each $\eta_{i}^{\left(j\right)}$ with sample size k, where $\hat{\sigma_{e}}$ is used as a plug‐in parameter of $\sigma_{e}$. We obtain E($y_{ij}|\eta_{ij}$) by averaging over k, and consider $\left(E(y_{i}^{\left(1\right)}),E(y_{i}^{\left(2\right)}),\ldots ,E(y_{i}^{\left(m\right)})\right)$ the posterior predictive distribution $p(\tilde{y_{i}}|y)$ of the individual i. We use the posterior mean of the distribution $p(\tilde{y_{i}}|y)$ as the individual's prediction of height and weight.

#### 3.4.2 Population Parameter‐Based Prediction (Pred‐P)

When individual parameter information is unavailable, we estimate the parameters $\eta_{i}$ by directly simulating them from Equation (2) of the HLM. Specifically, we sample the parameters $\eta_{i}$ from a normal distribution characterized by the plug‐in estimates $\hat{\beta }$ and $\hat{\Omega }$:

$$\eta_{i}|\hat{\beta },\hat{\Omega }∼N(\hat{\beta },\hat{\Omega }).$$

This procedure generates a set of samples $\left(\eta_{1},\eta_{2},\ldots ,\eta_{n}\right)$ for i=1,…,n individuals. This sample can be interpreted as an approximation of the posterior distribution of η, given all the data, though it omits the uncertainty surrounding the parameters β and Ω.

We sample from the posterior predictive distribution $p(y_{i}^{\left(l\right)}|\eta_{i},{\hat{\sigma }}_{e},a_{t})$ for l∈(1,2,…,k) as follows:

$$y_{i}^{\left(l\right)}|\eta_{i},{\hat{\sigma }}_{e},a_{t}∼N(\eta_{i1}+\eta_{i2}a_{t},{\hat{\sigma }}_{e}),$$

and consider the expectations $\left(E(y_{i}^{\left(1\right)}),E(y_{i}^{\left(2\right)}),\ldots ,E(y_{i}^{\left(k\right)})\right)$ the posterior predictive distribution $p(\tilde{y_{i}}|y_{i})$ for each new individual i, for which posterior mean $E(\tilde{y_{i}}|y)$ is derived.

Uncertainty surrounding the estimation of β and Ω is not accounted for; however, uncertainty in the estimation of the distribution of η is addressed, although full posterior distributions for $\eta_{i}$ for each individual are not sampled.

#### 3.4.3 Combined Prediction (Pred‐C)

As we are interested in predicting height, weight and BMI for the whole Target Group that includes individuals within or outside the Contributing Group‐2021, it seems reasonable to use individual parameter information when possible, thus combining Pred‐I and Pred‐P predictions. Table S6 in Supporting Information H lists the parameters used for different individuals in this approach. Generally, the Pred‐C method uses Pred‐I on individuals from Contributing Group‐2021 and Pred‐P otherwise.

### 3.5 Methods to Check the Prediction Accuracy

This study we emphasizes multiple prediction evaluations, as we believe that a diverse set of prediction summaries provides a more comprehensive view of the model's predictive capabilities. Our primary focus is on assessing predictions for an entire population of children, with a secondary interest in evaluating predictions for individual children. For each child, we use the posterior predictive mean as a summary of their posterior predictive distribution.

We focused mainly on the bivariate HLM's out‐of‐sample prediction accuracy.^5^ The univariate and bivariate checks include graphical summaries of posterior densities, summary statistics and statistical measures comparing predicted and observed distributions, such as Wasserstein distance and Euclidean distance.

#### 3.5.1 Within‐Sample Checks

Earlier, we had evaluated the model outcome for the Contributing Group with within‐sample posterior predictive checks appropriate for Bayesian HLMs.^6^ In this study, we compared the unconditional distribution parameters defined in Section 3.3 with the Contributing Group's empirical distribution parameters. The comparison acts as an additional within‐sample check, as it inspects our model's ability to provide the accurate unconditional distribution for height and weight.

#### 3.5.2 Out‐of‐Sample Checks

In addition to evaluating of the prediction accuracy of the model for the Target Group, we explored how measurements of the Contributing Group‐2021 act in the prediction, and examined prediction for ths group separately. The prediction method Pred‐I provides predictions only for the members of the Contributing Group‐2021 based on their individual parameters whereas Pred‐C provides predictions for the Target Group.

##### 3.5.2.1 Predictions for the Target Group

For out‐of‐sample checks, we provided visualizations for marginal densities and bivariate densities of the predictions compared to the Target Group's observations. To summarize the comparison between predicted and observed height and weight densities, we calculated first order Wasserstein distances. The general idea of Wasserstein distance is to estimate the minimum “cost” of transporting density mass A to density mass B. Wasserstein distance $W_{p}$ for probability masses X and Y is defined as

Wp(X,Y)=infX∼μY∼ν𝔼[d(X,Y)]p1/p

where μ and ν are the marginal distributions of X and Y, p≥1, and d(X,Y) is the ground cost of transporting a mass unit between two points x∈X and y∈Y, defined for p=1 as $d(X,Y)=||X-Y|{|}^{1}$. Infimum is taken over all the sets of joint distributions (X,Y) with marginal distributions of X and Y [27, 28].

The distances were calculated with the R package transport where the cost optimization algorithm is the default networkflow, which is a special form of a simplex algorithm that solves the optimal transportation problem of finding the minimum distance between the two distribution masses [29].

There were several reasons for choosing the Wasserstein distance over other statistical distance measures when comparing posterior predictive distributions with a reference distribution. First, in contrast to metrics such as the Euclidean distance, the Wasserstein distance evaluates the overall distance between two densities, rather than comparing predefined pairs of points. Given that some of our prediction approaches treat each child's growth parameters as interchangeable, the flexibility of Wasserstein distance is needed. Second, the Wasserstein distance can be calculated even when the supports of the compared distributions differ. Unlike other metrics, it considers not only the shape of the distribution but also its location relative to a reference distribution.

Below, we give the definition for the global mean and variance for each age year $a_{t}$ over Target Group's predictions and discuss some limitations of our approach. Denote the posterior predictive expectation of each individual of the target group by $m_{i}(y,a_{t})=E({\tilde{y}}_{i}|y)$ and the variance by $v_{i}(y,a_{t})$, i=1,…,n. These quantities correspond to the sample mean and variance. Then we define the posterior predictive mean of the Target Group as

$$E(m_{i}(y,a_{t}))=\overset{n}{\underset{i=1}{\sum }}m_{i}(y,a_{t})/n$$

and the variance as

$$Var(m_{i}(y,a_{t}))=\overset{n}{\underset{i=1}{\sum }}{\left(m_{i}(y,a_{t})-\bar{m}\right)}^{2}/(n-1).$$

This expression gives the between‐individual variation in the prediction. As within‐individual variation $\sum_{i}v_{i}(y,a_{t})$ is not added to the above definition, some of the variability is lost in the estimation of the total variability of the Target Group's measurements. This holds true for all prediction approaches.

##### 3.5.2.2 Predictions for the Contributing Group‐2021

We also investigated whether individual parameter information helps in predicting height and weight for each child in the Contributing Group‐2021. To address the individual‐level prediction capacity, measurements versus individual outcomes were plotted, and Euclidean distances between individuals' measurements and their predictions were calculated. For individual i's observation $\left(x_{h1},x_{w1}\right)$ and its corresponding prediction $\left(x_{h2},x_{w2}\right)$ the distance would be defined as $\sqrt{{\left(x_{h2}-x_{h1}\right)}^{2}+{\left(x_{w2}-x_{w1}\right)}^{2}}$. Additionally, we evaluated the predictions by treating the collection of observations as a density, ignoring individual‐level predictions. For this density‐wise comparison, we used Wasserstein distances.

#### 3.5.3 Simulations for Evaluation of Uncertainty in the Prediction Draws

In order to quantify the uncertainty in the prediction draws, we simulated 100 Pred‐P and Pred‐C method predictions $E({\tilde{y}}_{i}|y,a_{t})$ for each height and weight measurement of the Target Group.

We calculated expectations of summary statistics over the simulations as well as standard deviations over the expectations to quantify the variability across simulations. The Wasserstein and Euclidean distances were also averaged over the simulations. The figures in Section 4 are illustrative and are based on a randomly chosen simulation sample. We ensured that the figures provide the same conclusions, regardless of the simulation.

#### 3.5.4 Clinically Meaningful Differences Between Prediction and Outcome

When comparing summary statistics, it is important to define a clinically meaningful magnitude of differences to understand whether the prediction accuracy is coherent considering the study question, clinical relevance and subject matter. Here we propose criteria to define clinically meaningful differences in population‐level prediction based on summary statistics. We concentrate on the differences of predicted and true medians by age, although similar assessment can be performed with regards to other summary statistics.

We suggest that the difference in the median weight of at least 1 kg is meaningful in prediction, as we observed that between ages 2 and 11, the median annual weight gain ranges from 2 to 4 kg in the Contributing Group (Table S5 in Supporting Information G). Additionally, it is reported that the deviation of day‐to‐day weight measures is 1 to 2 kg in adults in Bhutani et al. [30]. Therefore, a prediction difference of ≤1kg is less than weight increase per year and the lower limit of adult weight fluctuation. The height measurements in the data were given in cm (without decimals), and hence, an absolute difference between predicted and observed heights of 1–2 cm may be due to rounding. Thus, the absolute difference in at least 2 cm of median height could be considered meaningful in prediction as it is more than a potential rounding error. The absolute difference in median BMI of 0.5 ${\text{kg/m}}^{2}$ or less can be considered meaningful, as median BMI changes in decimals over years, compared to height and weight.

## 4 Results

Below, the results for posterior unconditional distribution of height, weight and BMI for the Contributing Group are shown in addition to the results of the out‐of‐sample prediction for the Target Group and Contributing Group‐2021 (members of both Contributing Group and Target Group).

### 4.1 Unconditional Distributions of Height, Weight and BMI of the Contributing Group

Based on Table 2, only the standard deviation of the BMI differs by more than approximately one unit at largest, at the age of nine. The differences between standard deviations in other entities are smaller. In height, the difference between unconditional and observed mean is 1–2 cm at ages of 6, 8, 9, and 11; all other ages, it is smaller. In weight, the difference between unconditional and observed mean is approximately 1 kg at age of 11, while at the remaining ages, it is smaller.

**TABLE 2:** Within‐sample predictive check for posterior unconditional distribution of height, weight and BMI (Contributing Group measurements).

	Height (cm)		Weight (kg)		BMI (${\text{kg/m}}^{2}$)	
Age	Posterior	Observed	Posterior	Observed	Posterior	Observed
2	91.1 (4.4)	90.4 (4.5)	13.6 (1.6)	13.5 (1.7)	16.4 (1.4)	16.5 (1.2)
3	97.9 (4.4)	98.3 (4.6)	15.6 (1.9)	15.7 (2.0)	16.2 (1.4)	16.2 (1.2)
4	105.2 (4.6)	105.0 (4.7)	17.8 (2.3)	17.8 (2.4)	16.1 (1.5)	15.9 (1.3)
5	113.0 (5.2)	113.0 (5.0)	20.3 (2.8)	20.3 (3.0)	15.9 (1.6)	15.9 (1.5)
6	122.2 (5.5)	120.0 (5.4)	24.0 (4.0)	23.4 (3.8)	16.0 (1.9)	16.0 (1.7)
7	127.3 (5.6)	127.0 (5.5)	27.0 (4.6)	26.9 (5.0)	16.6 (2.1)	16.5 (2.3)
8	132.6 (5.9)	134.0 (5.8)	30.3 (5.5)	30.6 (6.1)	17.2 (2.4)	17.1 (2.6)
9	138.1 (6.2)	139.0 (6.0)	34.2 (6.7)	34.3 (7.3)	17.8 (2.7)	17.6 (3.8)
10	144.0 (6.5)	144.0 (6.5)	38.5 (8.2)	38.5 (8.8)	18.5 (3.1)	18.4 (3.2)
11	150.0 (7.0)	149.0 (6.9)	43.4 (10.1)	42.5 (10.5)	19.2 (3.5)	19.0 (3.7)

*Note:* Posterior and observed mean and standard deviation (SD) of the distribution are shown.

### 4.2 Out‐of‐Sample Prediction for Target Group: Marginal Distributions

Table 3 presents the main summary statistics for the empirical distribution of the Target Group's observations and predictions based on the Pred‐P and Pred‐C methods. Averages over the prediction simulations (described in Section 3.5.3), are provided, with standard deviations across the simulations available in the Table S4 of the Supporting Information D [24]. Outcomes of both methods resemble the distribution of the observations better at younger ages in weight and BMI. The differences between Pred‐P and Pred‐C prediction accuracy for means and medians are inconclusive. For height, the largest difference between predicted and observed medians is 2 cm at the age of six; for weight, 2 kg at the age of 11; and in BMI, 0.3 ${\text{kg/m}}^{2}$ (not visible from the table) at the age of 11. Based on our definition of clinically meaningful differences between the prediction and outcome, our predictions do not differ from the reference in a clinically relevant manner except for the above‐described ages and units.

**TABLE 3:** Prediction statistics, compared with summary statistics of Target Group's empirical distribution of the observations.

		Mean (SD)			Median ($q_{05}$, $q_{95}$)			Skewness (kurtosis)		
	Age	Observed	Pred‐P	Pred‐C	Observed	Pred‐P	Pred‐C	Observed	Pred‐P	Pred‐C
Height (cm)	4	105 (4)	105 (4)	105 (4)	105 (98,113)	105 (99,112)	105 (99,111)	0.3 (3.2)	0.1 (3.1)	0.3 (3.3)
	5	112 (5)	113 (5)	113 (4)	112 (105,120)	113 (106,121)	113 (106,120)	0.1 (3)	0.1 (3)	0.4 (3.3)
	6	120 (5)	122 (5)	122 (5)	120 (112,129)	122 (114,131)	121 (114,130)	0.2 (3.2)	0.1 (3)	0.3 (3.2)
	7	127 (6)	127 (5)	126 (5)	127 (118,136)	127 (119,136)	126 (118,136)	0.1 (3)	0.1 (3)	0.4 (3.5)
	8	134 (6)	133 (5)	132 (5)	133 (124,144)	132 (124,142)	132 (124,141)	0.2 (3.2)	0.1 (3)	0.1 (2.8)
	9	138 (6)	138 (6)	137 (6)	138 (129,148)	138 (129,148)	137 (128,146)	0 (2.8)	0.1 (3)	0.1 (2.9)
	10	144 (6)	144 (6)	144 (6)	144 (134,154)	144 (134,154)	144 (135,155)	0.2 (3)	0.1 (2.9)	0.2 (3)
	11	149 (7)	150 (7)	151 (7)	149 (137,160)	150 (139,161)	151 (140,162)	0.4 (3.3)	0.1 (3)	0.2 (2.8)
Weight (kg)	4	18 (2)	18 (2)	18 (2)	18 (15,22)	18 (15,21)	18 (15,21)	0.8 (4)	0.4 (3.3)	0.6 (4)
	5	20 (3)	20 (3)	20 (3)	20 (16,26)	20 (16,25)	20 (17,25)	1 (4.9)	0.4 (3.1)	0.9 (4.6)
	6	23 (4)	24 (4)	23 (3)	23 (19,31)	24 (18,30)	23 (19,29)	1.6 (8.3)	0.5 (3.3)	1 (5.8)
	7	27 (5)	27 (4)	26 (4)	26 (21,35)	27 (20,35)	25 (21,33)	1.7 (8.1)	0.5 (3.4)	1.3 (5.9)
	8	31 (6)	30 (5)	30 (5)	30 (24,45)	30 (23,39)	29 (24,40)	1.5 (5.9)	0.5 (3.5)	1.4 (5.9)
	9	34 (7)	34 (6)	34 (6)	33 (26,48)	34 (25,45)	33 (26,45)	1.2 (4.8)	0.5 (3.6)	1.3 (5.8)
	10	40 (9)	38 (8)	40 (9)	38 (29,57)	38 (27,53)	38 (29,58)	1.5 (6.8)	0.6 (3.6)	1.5 (6.3)
	11	42 (10)	43 (10)	44 (10)	40 (30,60)	42 (29,61)	42 (32,63)	1.1 (4.7)	0.7 (3.8)	1.2 (4.9)
BMI (${\text{kg/m}}^{2}$)	4	16 (1)	16 (1)	16 (1)	16 (14,18)	16 (14,18)	16 (15,18)	0.6 (4.2)	0.2 (3.1)	0.3 (3.7)
	5	16 (2)	16 (1)	16 (1)	16 (14,19)	16 (14,18)	16 (14,18)	1.3 (6.8)	0.2 (3)	0.6 (4.3)
	6	16 (2)	16 (2)	16 (1)	16 (14,19)	16 (13,19)	16 (14,18)	1.7 (7.9)	0.3 (3.1)	0.9 (6)
	7	16 (2)	17 (2)	16 (1)	16 (14,20)	16 (14,20)	16 (14,19)	2.1 (10.9)	0.3 (3.2)	1.1 (5.9)
	8	17 (3)	17 (2)	17 (2)	17 (14,23)	17 (14,21)	17 (15,21)	1.7 (6.2)	0.4 (3.2)	1.7 (7.6)
	9	18 (3)	18 (3)	18 (2)	17 (15,24)	18 (14,22)	17 (15,22)	1.4 (5.3)	0.4 (3.3)	1.5 (7.2)
	10	19 (4)	18 (3)	19 (3)	18 (15,25)	18 (14,24)	18 (15,25)	1.5 (6.1)	0.5 (3.3)	1.6 (6.9)
	11	19 (3)	19 (3)	19 (3)	18 (15,25)	19 (14,25)	18 (15,26)	1.2 (4.6)	0.5 (3.4)	1.2 (4.8)

*Note:* The abbreviation Pred‐P stands for population parameter‐based prediction, and Pred‐C stands for combined prediction. The prediction summary statistics are averaged over the prediction simulations.

It is noticeable that the prediction range is wider in Pred‐P method‐based predictions compared to the Pred‐C, where the range is slightly underestimated. Based on Table 3, the distribution of predictions from the Pred‐C method is more closely aligned with the shape and location of the empirical density of the Target Group's observations. However, the Pred‐C distribution has a slightly narrower value range than the empirical distribution of the observations, pointing towards underestimation of the scale parameter.

**FIGURE 2:**
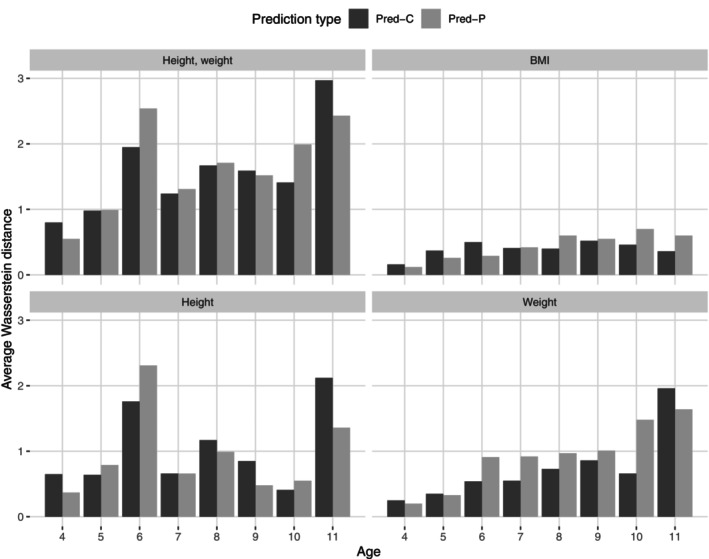
Average Wasserstein distances by age for bivariate predictions (height, weight, and BMI) using the population parameter‐based method (Pred‐P) and the combined method that integrates individual parameters when available (Pred‐C). The distances are calculated by comparing the predictions against the Target Group's observations (defined in Section 2). Dark grey colour corresponds to Pred‐C whereas pale grey to Pred‐P outcomes.

Figure 2 shows the average Wasserstein distances between the marginal and bivariate predictions and the measured observations of the Target Group. Particularly for weight and BMI prediction for older ages, the Pred‐C method outperforms the Pred‐P method. The results are inconclusive for height. This result should be considered together with Table 3, where skewness and kurtosis of the Pred‐C distribution are considerably closer to the observed measurements of weight and BMI, compared to the Pred‐P distribution.

### 4.3 Out‐of‐Sample Prediction for Bivariate Outcomes

**FIGURE 3:**
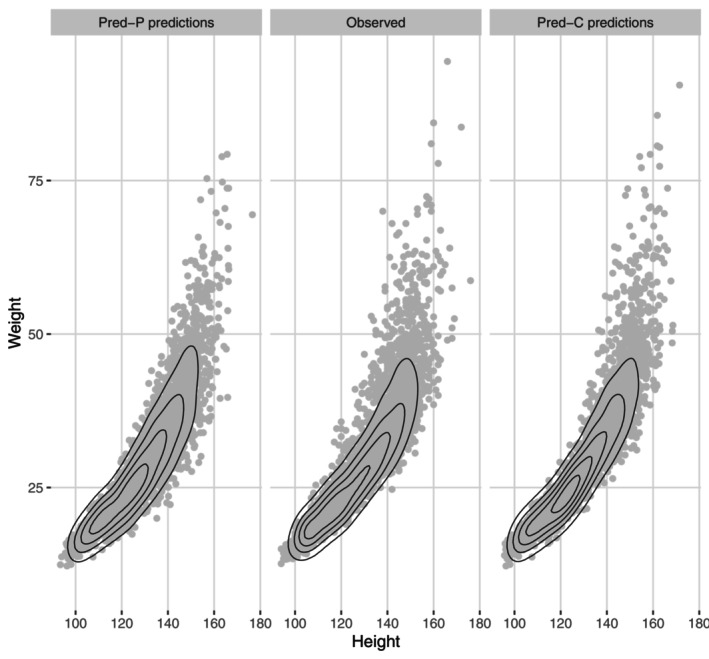
Joint height and weight predictions from the population parameter–based (Pred‐P) and combined (Pred‐C) methods, the latter using individual parameter information when available. Predictions are compared to observations of the Target Group, denoted here as “Observed”. (Target Group is defined in Section 2).

The main benefit of a bivariate model is its improved ability to predict height and weight outcomes together, compared to predicting height and weight separately based on univariate models.^7^ Figure 3 compares the joint densities of height and weight observations to the predictions of the Pred‐P and Pred‐C methods. The figure shows how for weights‐for‐heights, both distribution shape and extremely high observations are more accurately predicted by the Pred‐C method than the Pred‐P method.

We summarized the out‐of‐sample predictive performance of the Pred‐P and Pred‐C methods using Wasserstein distances. As shown in Figure 3, the average first‐order Wasserstein distances between height and weight bivariate predictions and the Target Group's observations were smaller for the Pred‐C method. The exceptions were ages 4, 9, and 11. The differences between the two methods were relatively small. In general, the Wasserstein distances increase with age for all predicted quantities except height. The standard deviations for the average Wasserstein distances across prediction simulations were ≈0.1‐0.2 for the Pred‐P method and two times smaller for the Pred‐C method, which implies that using individual parameters provides more consistent prediction draws. The standard deviations for Wasserstein distances across prediction simulation draws are given in Table S3 of the Supporting Information D [24].

### 4.4 Prediction for Contributing Group‐2021

We investigated possible benefits of using individual parameter information in predicting height and weight for each child in the Contributing Group‐2021 (members of both Contributing Group and Target Group). The focus was on assessing predictions both at the individual level and for the entire group of children.

For individual‐level predictions, Figure S1 in Supporting Information F provides averages of Euclidean distances for Contributing Group‐2021's observations versus the Pred‐I and Pred‐P predictions. The distances were consistently smaller for the Pred‐I method, compared to Pred‐P. Figure S2 in Supporting Information F shows how the Pred‐I method affects predictions against the Contributing Group‐2021's observed height, weight and BMI, compared to the Pred‐P method. We see that Pred‐I is more accurate than Pred‐P for all three entities when predictions are compared pairwise for each individual. However, it can still be seen that the prediction for children with higher weights and BMIs tend to underestimate the values.

For the population of Contributing Group‐2021 individuals, predictions for ages 7–9 are shown to illustrate the difference between Pred‐I versus Pred‐P‐based predictions (Figure S3 in Supporting Information F). The Pred‐I method provides narrow densities (in weight) compared to the observed heights and weights, but more closely resembles the observed densities in shape. The Wasserstein distances provided in Figure S1 in Supporting Information F for density‐wise comparison show that Pred‐P fared better than Pred‐I at ages of 4, 7 and 11, and otherwise the predictions based on the Pred‐I method were closer to the observations.

## 5 Discussion

We showed that the bivariate Bayesian HLM used in our study allows for clinically meaningful predictions for the out‐of‐sample population. We also propose feasible methods for evaluating the predictive accuracy of the model. While predictions based solely on population‐level parameters provided reasonable accuracy, incorporating individual‐level parameters resulted in improvements, particularly for more skewed distributions. This suggests that individual‐level information should be considered in population‐level predictions, when available. Below, we discuss the results of our study in more detail.

### 5.1 Correlation of Height and Weight

Correlation between height and weight is a topic that has been extensively studied. However, some answers remain partially missing due to the lack of longitudinal data spanning wide age ranges and time periods to reveal secular changes in growth patterns [31]. By utilizing a nationwide register dataset over multiple years with our HLM, we presented the correlation between weight and height over time.^8^ We were able to derive accurate parameter estimates for the unconditional distribution of BMI with the correlation estimates of height and weight (Table 2).

The effect of regression to the mean in terms of growth is reported before, that is, taller children grow slower than others [32]. With regard to weight, we observed a reverse effect, that heavier children gained weight quicker than their counterparts in 6–11 years of age.^9^ This age range typically corresponds to the primary school age in Finland. Aris et al. reported that heavier children would grow more in height (until puberty age) [31], but our findings based on the limited dataset of this study do not support this and point to a direction for further investigation. A natural follow‐up study would be wider analysis of the growth trends in Finnish children.

### 5.2 Benefits and Challenges of Using Individual‐Level Information in Prediction

We showed that bivariate HLMs provide reasonable and clinically meaningful within‐sample and out‐of‐sample predictive accuracy in terms of summary statistics. Only the upper tails of weight and BMI were incompletely captured. Nevertheless, some accuracy increase from utilization of individual‐level information was observed (Table 3). This may be because heavier children tend to gain weight more rapidly than their counterparts at ages 6–11 (See Supporting Information C), a pattern that individual‐level parameters may describe more accurately than population‐level parameters.

The summary statistics, visual inspection and statistical distance calculations showed that using the individual‐level parameters, when possible (Pred‐C) instead of population‐level parameters only (Pred‐P) improves prediction for the target population (Figures 2 and 3) especially in terms of distribution skewness and kurtosis. As those two distribution moments increase upon age in weight, weight prediction benefits substantially from using individual parameter information in prediction. In terms of bivariate prediction, using individual parameter information provided a moderate advantage for most ages.

An additional advantage of using individual‐level parameters is the reduced variability in predictions across prediction draws, in contrast to predictions based on population‐level parameters that exhibit greater variability. This is shown in Table S3 Supporting Information D [24], where the reported standard deviations are based on the prediction draw simulations. This effect arises because each individual has a posterior predictive distribution for their own growth parameters. In contrast, sampling individual‐level parameters from Equation (2) results in a larger range of possible individual parameter values.

At the same time, the standard deviation of the prediction density decreased more when using individual‐level parameters in prediction (Table 3). However, shrinkage towards the mean is visible in population parameter‐based prediction as well. This behaviour is typical of hierarchical linear models that pool predictions towards the centre. The shrinkage issue could be addressed by defining a mixture distribution for latent variables such as height or weight, or by using a t‐distribution instead of a normal distribution in the hierarchical linear model.

Furthermore, we used the posterior predictive mean $E(p({\tilde{y}}_{i}|y,a_{t}))$ for each individual at age $a_{t}$ as a summary measure of the prediction. Some of the variability may have been reduced, potentially narrowing the prediction range. It should also be noted that we cannot assume independence of the posterior means due to the conditioning on the data. This implies that the variance estimate for the target population's predictions may require a covariance term.

If the focus is on predicting the growth of each child with using individual growth parameters for individuals with previously available growth data only (Pred‐I), incorporating individual‐level information in prediction offers significant benefits and is overall accurate compared to population‐parameter‐based predictions for the same group (Figures S1 and S3 in Supporting Information F). However, when assessing growth predictions at the population level, the use of individual‐level information does not always provide an additional benefit (Figures S1 and S2 in Supporting Information F). This surprising result may be partly due to the study design, where most individuals had only two observations, which was the minimum requirement for inclusion in the Contributing Group.^10^ It is well known that such a study design is suboptimal for investigating individual growth [33], limiting the precision of individual growth parameter estimates.

Based on this, we conjecture that a minimum of more than two measurements per individual would lead to more precise individual parameter estimates, resulting in increased accuracy of the corresponding prediction approaches. However, it is known that as little as two observations per individual suffice to contribute properly to population parameter‐based estimates (see Gelman et al. [25, pp. 275–276]). This justifies the inclusion of individuals with only two observations, as excluding them would result in a significant loss of population‐level information.

### 5.3 Dataset and Applications for the Prediction Framework

To save computation time, the analysed dataset was restricted to three neighbouring municipalities in southern Finland, where individuals are comparable to Finnish children in terms of age‐adjusted BMI [19]. Two age groups, 2–5 and 6–11 years of age, were modelled separately because compared to 2–5 year olds, 6–11 year olds had longer and heavier tails in their respective distributions of weights (Figure 1) and we envisaged that the start of school (in Finland at the age of 6–7) might impact the development of weight and also change lifestyle. We focused on modelling observations obtained before puberty, as the coverage of measurements decreases with age.

The Finlapset register provides sufficient information for nationwide or even region‐wide studies of contemporary childhood growth in Finland. Such datasets, if of high quality, should be utilized when available, as they cover the population without the imbalance issues common in survey datasets. The framework we have described can be applied to predict height, weight, and BMI for the entire Finnish population. Notably, a practical consideration when applying bivariate HLMs to larger register datasets is the required computational time, which may limit the model's usefulness for inference based on larger populations. In order to increase the computation speed, Cholesky decomposition of the covariance matrix Ω could be examined, along with further vectorisation of the Stan model components.

Our model does not require observations to be independent, although independence is assumed given the individual random effects. While growth at the individual level is diverse, we believe that the common biological background should not be disregarded. The strength of HLMs lies in their ability to account for population‐wide trends, in contrast to the no‐pooling approach often used in longitudinal settings. The partial pooling approach also makes sense from a biological perspective, highlighting its potential for application in epidemiological settings.

Bivariate longitudinal data arise in certain outcomes such as systolic and diastolic blood pressure measured over time, eye or ear function, and functional measurements of symmetric organs like muscles, kidneys, or lungs. The model developed here can be adapted to include both time‐invariant and time‐varying covariates for prediction.

The model validation techniques employed in this study, such as Wasserstein and Euclidean distances, effectively supported the comparative assessment of different prediction models. While additional metrics such as Kullback—Leibler divergence were considered, they were not implemented due to mismatches between the support of predicted and observed distributions, and because the primary objective was to evaluate predictions at the population level, with individual‐level accuracy being secondary.

Both Wasserstein and Euclidean distances are known to be sensitive to outliers. However, in this context, the impact of the outliers on the distances was minimal: diagnostic plots and summary statistics showed that the predicted and reference densities were closely aligned (Table 3, Figure 2). The discrepancies observed were more indicative of under‐dispersion in the predictive distributions rather than extreme values, as explained in Section 4.2. Nonetheless, in settings with heavy‐tailed or skewed data, the sensitivity of these metrics to outliers warrants careful consideration.

### 5.4 Improvements for the Prediction Approaches of This Study

Many ideas on the nature of growth and its longitudinal modelling, which are relevant to this project, are presented in the works of Ying Wei and colleagues, who have also used Finnish growth cohort data as an example in two works by Wei et al. [32, 34]. The challenges with tail prediction lead us to consider alternative approaches to joint modelling, such as bivariate quantile regression models, which may better capture the increasing skewness in weight, extending the model proposed by Wei et al. [32].

Another approach would be to extend our model across all ages and allow the variance‐covariance matrix to depend on age. This would yield models that provide more accurate predictions and illustrate how growth patterns change across birth cohorts over time. In longitudinal univariate childhood growth modelling, more complex non‐linear functions are typically advised to use instead of linear regression models [1]. The cost‐benefit ratio in utilizing more complex growth functions may not be favourable when applying bivariate modelling to large datasets with typically only a few measurements per individual. However, this balance may shift when predicting growth over a wide age range with numerous measurements per individual.

There are several approaches to define individual‐level prediction with incorporation of the individual's observations in the prediction, such as the random effects model‐based approach described by Diggle [35, chapter 5, p. 110], or the prediction method II described by Fearn [26] for Bayesian hierarchical models. We provide a definition for such type of prediction given our model in Supporting Information B [24]. Comparing these methods with the prediction methods presented in this paper could yield valuable insights.

An important benefit of the Bayesian approach is its ability to provide predictions based on the expectations of the posterior predictive distribution for each individual. We utilized this benefit only partially due to practical considerations. However, exploring a fully Bayesian framework that accounts for hyperparameter uncertainty in population‐level predictions may further improve prediction accuracy. Additionally, future studies could benefit from using informative priors, rather than semi‐informative or non‐informative ones, by integrating findings from existing research on the same topic.

## Author Contributions

**Tuuli Kauppala:** conceptualization, analysis, methodology, writing – original draft (lead) and writing – review and editing. **Tuomo Susi:** conceptualization (supporting), writing – review and editing. **Sangita Kulathinal:** conceptualization, methodology, supervision, writing – original draft (supporting) and writing – review and editing.

## Funding

The authors have nothing to report.

## Conflicts of Interest

The authors declare no conflicts of interest.

## Supporting information




**Data S1.** Supporting Information.

## Data Availability

Unfortunately, the data are not publicly available due to privacy restrictions, as they contain sensitive medical information. The Stan code for the HLM is shared in the Supporting Information E.

## ENDNOTES

^1^
As outlined in Kauppala [12, p. 13].

^2^
Table S5 in Supporting Information G summarizes the key measurement statistics for children aged 2–11 years in the Contributing Group.The summary frequencies for the number of observations per individual are provided in Kauppala [12, p. 15, tab. 3].

^3^
The Lewandowski‐Kurowicka‐Joe (LKJCorr) correlation prior is defined in most detail in Gelman et al. [21, p. 579].

^4^
The computation and convergence checks are described in more detail in Kauppala [12, pp. 28–30].

^5^
Based on within‐sample posterior predictive checks in Kauppala [12], we believed that the prediction accuracy of the bivariate HLMs were better than the corresponding univariate case, especially for BMI.

^6^
Described in Kauppala [12, pp. 31–33].

^7^
Shown by within‐sample checks in Kauppala [12, p. 37].

^8^
In Kauppala [24], it was observed that between‐person variation in height and weight is considerably larger than within‐person variation, especially in weight (Model estimates based on the work given in Supporting Information C).

^9^
In Supporting Information C, the parameter estimates $\rho_{W_{0},W_{1}}$ can be interpreted as between‐subject level correlation terms. More details in Kauppal [12, p. 43 and 47].

^10^
The modelling dataset description is provided in Kauppala [12, p. 15].
