# Supplementary material for: Modelling and Predicting Population‐Level Growth With Individual‐Level Information
Source: Stat Med. 2026 Feb 22;45(3-5):e70421. doi: 10.1002/sim.70421 (PMC12926727; doi:10.1002/sim.70421)
Supplement: Supplementary file 1 — Data S1. Supporting Information. [file SIM-45-0-s001.pdf]

# 1 | SUPPLEMENTARY MATERIAL A

The standard log-normal theory states: if $\boldsymbol{X} = (X_1, X_2)$ has bivariate log-normal distribution so that $\boldsymbol{Y} = \log(\boldsymbol{X})$ has bivariate normal distribution with mean $\boldsymbol{\mu} = (\mu_1, \mu_2)$, and variance-covariance matrix $\Sigma = ((\sigma^2_{ij})), i = 1, 2, j = 1, 2$, and $\sigma_{12} = \sigma_{21}$ then

$$E(X_j) = e^{(\mu_j + \sigma^2_{jj}/2)},$$
$$Var(X_j) = e^{(2\mu_j + \sigma^2_{jj})}(e^{\sigma^2_{jj}} - 1),$$
$$cov(X_1, X_2) = e^{(\mu_1 + \mu_2 + (\sigma^2_{11} + \sigma^2_{22})/2)}(e^{\sigma^2_{12}} - 1).$$

See Feng et al (2013)[1]

# 2 | SUPPLEMENTARY MATERIAL B

The joint distribution of $(\mathbf{y}_i, \boldsymbol{\eta}_i)$ for given age $a_t$, and $\boldsymbol{\beta}$ and $(\boldsymbol{\sigma}^2_e, \Omega)$ is a 6– variate normal distribution with mean vector $(\boldsymbol{\mu}_y, \boldsymbol{\mu}_\eta)$ and variance-covariance matrix $\Sigma = (\Sigma_{yy}, \Sigma_{y\eta}; \Sigma_{\eta y}, \Sigma_{\eta\eta})$. As shown earlier, $\boldsymbol{\mu}_{y_t} = E(\mathbf{Y}_{ti}) = \boldsymbol{\beta}_0 + \boldsymbol{\beta}_1 a_t$ and $\Sigma_{yy} = \boldsymbol{\sigma}^2_e + \mathbf{U}\Omega\mathbf{U}^t$, where $U = (1, 0, a_t, 0; 0, 1, 0, a_t)$ is a $2 \times 4$ matrix. The covariance matrix $\Sigma_{y\eta} = \Sigma^t_{\eta y}$ can be easily shown to be $\mathbf{U}\Omega - \boldsymbol{\mu}_y \boldsymbol{\mu}_\eta$.

The conditional expectation and variance-covariance matrix of $\boldsymbol{\eta}_i$ given $\mathbf{y}_i$ and age $a_t$, and $\boldsymbol{\theta} = (\boldsymbol{\beta}, \boldsymbol{\sigma}^2_e, \Omega)$ are:

$$E(\boldsymbol{\eta}_i | \mathbf{y}_i, a_t, \boldsymbol{\theta}) = \boldsymbol{\mu}_\eta + \Sigma_{\eta y}\Sigma^{-1}_{yy}(\mathbf{y}_i - \boldsymbol{\mu}_{yt})$$
$$Var(\boldsymbol{\eta}_i | \mathbf{y}_i, a_t, \boldsymbol{\theta}) = \Sigma_{\eta\eta} - \Sigma_{\eta y}\Sigma^{-1}_{yy}\Sigma_{y\eta}.$$

Note that we have used data $\mathbf{Y}_{ti}$ for individual $i$ collected at age $a_t$. The above results can be extended easily to include all data from individual $i$ collected at different ages.

# 3 | SUPPLEMENTARY MATERIAL C

Growth parameters in Tables S1 and S2, as described in Kauppala (2021) , pages 42 and 68.[2]

**TABLE S1 Growth parameters based on the bivariate, unconditional HLM.** Parameter estimates and other statistics for bivariate, unconditional hierarchical linear model.

| **Males, 2-6 year olds** | | | | | |
|---|---|---|---|---|---|
| Parameter | Mean | SE | 10% CI | 90% CI | $\hat{R}$ |
| $\beta_{H_0}$ | 4.51 | 5e-05 | 4.51 | 4.51 | 1.01 |
| $\beta_{W_0}$ | 2.61 | 1e-04 | 2.61 | 2.61 | 1.01 |
| $\beta_{H_1}$ | 0.07 | 1e-05 | 0.07 | 0.07 | 1.00 |
| $\beta_{W_1}$ | 0.13 | 2e-05 | 0.13 | 0.13 | 1.00 |
| $\sigma_{e_H}$ | 0.02 | 6e-06 | 0.02 | 0.02 | 1.00 |
| $\sigma_{e_W}$ | 0.05 | 2e-05 | 0.05 | 0.05 | 1.00 |
| $\rho_{H_0,W_0}$ | 0.88 | 5e-04 | 0.87 | 0.89 | 1.01 |
| $\rho_{H_0,H_1}$ | -0.45 | 1e-03 | -0.49 | -0.42 | 1.00 |
| $\rho_{H_0,W_1}$ | -0.30 | 1e-03 | -0.34 | -0.26 | 1.00 |
| $\rho_{W_0,H_1}$ | -0.36 | 1e-03 | -0.40 | -0.32 | 1.00 |
| $\rho_{W_0,W_1}$ | -0.16 | 1e-03 | -0.20 | -0.12 | 1.00 |
| $\rho_{H_1,W_1}$ | 0.97 | 1e-03 | 0.97 | 0.98 | 1.05 |
| $\sigma_{H_0}$ | 0.04 | 2e-05 | 0.04 | 0.04 | 1.00 |
| $\sigma_{W_0}$ | 0.11 | 5e-05 | 0.11 | 0.12 | 1.00 |
| $\sigma_{H_1}$ | 0.01 | 1e-05 | 0.01 | 0.01 | 1.00 |
| $\sigma_{W_1}$ | 0.29 | 3e-05 | 0.03 | 0.03 | 0.99 |

**TABLE S2 Growth parameters based on the bivariate, unconditional HLM.** Parameter estimates and other statistics for bivariate, unconditional hierarchical linear model.

| **Males, 6-12 year olds** | | | | | |
|---|---|---|---|---|---|
| Parameter | Mean | SE | 10% CI | 90% CI | $\hat{R}$ |
| $\beta_{H_0}$ | 4.80 | 4e-05 | 4.80 | 4.81 | 1.01 |
| $\beta_{W_0}$ | 3.16 | 1e-04 | 3.16 | 3.17 | 1.00 |
| $\beta_{H_1}$ | 0.04 | 9e-06 | 0.04 | 0.04 | 1.01 |
| $\beta_{W_1}$ | 0.12 | 2e-05 | 0.12 | 0.12 | 1.01 |
| $\sigma_{e_H}$ | 0.01 | 6e-06 | 0.01 | 0.01 | 1.01 |
| $\sigma_{e_W}$ | 0.05 | 2e-05 | 0.05 | 0.05 | 1.00 |
| $\rho_{H_0,W_0}$ | 0.79 | 5e-04 | 0.78 | 0.80 | 1.00 |
| $\rho_{H_0,H_1}$ | -0.22 | 2e-04 | -0.25 | -0.18 | 1.00 |
| $\rho_{H_0,W_1}$ | -0.09 | 1e-03 | -0.11 | -0.06 | 1.00 |
| $\rho_{W_0,H_1}$ | -0.21 | 2e-03 | -0.25 | -0.18 | 1.00 |
| $\rho_{W_0,W_1}$ | 0.14 | 4e-04 | 0.11 | 0.17 | 1.00 |
| $\rho_{H_1,W_1}$ | 0.89 | 2e-03 | 0.88 | 0.91 | 1.03 |
| $\sigma_{H_0}$ | 0.04 | 3e-04 | 0.04 | 0.04 | 1.00 |
| $\sigma_{W_0}$ | 0.16 | 1e-03 | 0.15 | 0.16 | 1.00 |
| $\sigma_{H_1}$ | 0.00 | 2e-05 | 0.00 | 0.01 | 1.00 |
| $\sigma_{W_1}$ | 0.03 | 1e-04 | 0.03 | 0.03 | 1.00 |

## 4 | SUPPLEMENTARY MATERIAL D

Averaged Wasserstein distances with standard deviations for the prediction simulations in Table S3. We also present summary statistics with their standard deviations (SD) over prediction simulations in Table S4.

**TABLE** S3 Average Wasserstein distances and their standard deviations (SD) across the prediction simulation for bivariate population parameter-based (Pred-P) and individual parameter upon availability-based (Pred-C) predictions compared to the observed heights, weights and BMI of the Target Group. The smaller distance is bolded.

| | Wasserstein (height,weight) | | Wasserstein (height) | | Wasserstein (weight) | | Wasserstein (BMI) | |
|---|---|---|---|---|---|---|---|---|
| Age | Pred-P | Pred-C | Pred-P | Pred-C | Pred-P | Pred-C | Pred-P | Pred-C |
| 4 | 0.55 (0.08) | 0.8 (0.1) | 0.37 (0.1) | 0.65 (0.11) | 0.2 (0.07) | 0.25 (0.06) | 0.12 (0.04) | 0.16 (0.03) |
| 5 | 0.99 (0.18) | 0.98 (0.06) | 0.79 (0.21) | 0.64 (0.08) | 0.33 (0.07) | 0.35 (0.04) | 0.26 (0.03) | 0.37 (0.02) |
| 6 | 2.54 (0.24) | 1.95 (0.08) | 2.31 (0.23) | 1.76 (0.09) | 0.91 (0.11) | 0.54 (0.05) | 0.29 (0.04) | 0.5 (0.02) |
| 7 | 1.31 (0.2) | 1.24 (0.09) | 0.66 (0.22) | 0.66 (0.09) | 0.92 (0.15) | 0.55 (0.09) | 0.42 (0.05) | 0.41 (0.03) |
| 8 | 1.71 (0.17) | 1.67 (0.1) | 0.99 (0.21) | 1.17 (0.09) | 0.97 (0.1) | 0.73 (0.07) | 0.6 (0.06) | 0.4 (0.03) |
| 9 | 1.52 (0.12) | 1.59 (0.08) | 0.48 (0.11) | 0.85 (0.1) | 1.01 (0.17) | 0.86 (0.08) | 0.55 (0.06) | 0.52 (0.03) |
| 10 | 1.99 (0.24) | 1.41 (0.04) | 0.55 (0.19) | 0.41 (0.04) | 1.48 (0.32) | 0.66 (0.06) | 0.7 (0.08) | 0.46 (0.02) |
| 11 | 2.43 (0.33) | 2.97 (0.1) | 1.36 (0.26) | 2.12 (0.07) | 1.64 (0.31) | 1.96 (0.1) | 0.6 (0.09) | 0.36 (0.04) |

**TABLE** S4 Summary statistics with their standard deviations (SD) over prediction simulations. The abbreviation Pred-P stands for population parameter-based prediction, and Pred-C stands for combined prediction that uses individual parameters upon availability. The prediction summary statistics are averaged over the prediction simulations.

| | Age | Source | Mean (SD) | Median (SD) | SD (SD) | q05 (SD) | q95 (SD) | Skewness (SD) | Kurtosis (SD) |
|---|---|---|---|---|---|---|---|---|---|
| Height | 4 | Pred-C | 17.7 (0.1) | 17.5 (0.1) | 2 (0.1) | 14.7 (0.2) | 21.3 (0.3) | 0.6 (0.1) | 4 (0.5) |
| | 4 | Pred-P | 17.8 (0.1) | 17.6 (0.1) | 2.1 (0.1) | 14.6 (0.2) | 21.5 (0.2) | 0.4 (0.1) | 3.3 (0.4) |
| | 5 | Pred-C | 20.2 (0.1) | 20.1 (0.1) | 2.5 (0) | 16.8 (0.1) | 24.7 (0.1) | 0.9 (0.1) | 4.6 (0.2) |
| | 5 | Pred-P | 20.3 (0.1) | 20.1 (0.1) | 2.7 (0.1) | 16.2 (0.2) | 25 (0.3) | 0.4 (0.1) | 3.1 (0.3) |
| | 6 | Pred-C | 23.4 (0.1) | 23 (0.1) | 3.4 (0.1) | 18.8 (0.1) | 29 (0.2) | 1 (0.1) | 5.8 (0.3) |
| | 6 | Pred-P | 24 (0.2) | 23.7 (0.2) | 3.7 (0.1) | 18.4 (0.2) | 30.5 (0.4) | 0.5 (0.1) | 3.3 (0.4) |
| | 7 | Pred-C | 26 (0.1) | 25.3 (0.1) | 4 (0.1) | 20.9 (0.1) | 33.2 (0.5) | 1.3 (0.1) | 5.9 (0.4) |
| | 7 | Pred-P | 27 (0.2) | 26.6 (0.2) | 4.4 (0.2) | 20.4 (0.3) | 34.7 (0.5) | 0.5 (0.1) | 3.4 (0.4) |
| | 8 | Pred-C | 30.3 (0.1) | 29.3 (0.2) | 5.2 (0.1) | 24 (0.1) | 40.3 (0.2) | 1.4 (0.1) | 5.9 (0.2) |
| | 8 | Pred-P | 30.3 (0.2) | 29.9 (0.3) | 5.2 (0.2) | 22.5 (0.4) | 39.5 (0.7) | 0.5 (0.1) | 3.5 (0.5) |
| | 9 | Pred-C | 33.9 (0.1) | 32.8 (0.1) | 6.2 (0.1) | 26.1 (0.1) | 44.7 (0.6) | 1.3 (0.1) | 5.8 (0.3) |
| | 9 | Pred-P | 34.2 (0.3) | 33.6 (0.4) | 6.4 (0.3) | 24.7 (0.5) | 45.5 (0.8) | 0.5 (0.2) | 3.6 (0.7) |
| | 10 | Pred-C | 39.6 (0.1) | 38.2 (0.2) | 8.6 (0.1) | 29.1 (0.1) | 57.8 (0.3) | 1.5 (0) | 6.3 (0.2) |
| | 10 | Pred-P | 38.5 (0.4) | 37.7 (0.5) | 8 (0.3) | 27 (0.6) | 52.7 (1.1) | 0.6 (0.2) | 3.6 (0.6) |
| | 11 | Pred-C | 44 (0.1) | 41.7 (0.1) | 10.1 (0.1) | 31.6 (0.1) | 63 (0.3) | 1.2 (0) | 4.9 (0.1) |
| | 11 | Pred-P | 43.3 (0.4) | 42.3 (0.5) | 9.8 (0.4) | 29.3 (0.6) | 60.8 (1.3) | 0.7 (0.2) | 3.8 (0.6) |
| Weight | 4 | Pred-C | 17.7 (0.1) | 17.5 (0.1) | 2 (0.1) | 14.7 (0.2) | 21.3 (0.3) | 0.6 (0.1) | 4 (0.5) |
| | 4 | Pred-P | 17.8 (0.1) | 17.6 (0.1) | 2.1 (0.1) | 14.6 (0.2) | 21.5 (0.2) | 0.4 (0.1) | 3.3 (0.4) |
| | 5 | Pred-C | 20.2 (0.1) | 20.1 (0.1) | 2.5 (0) | 16.8 (0.1) | 24.7 (0.1) | 0.9 (0.1) | 4.6 (0.2) |
| | 5 | Pred-P | 20.3 (0.1) | 20.1 (0.1) | 2.7 (0.1) | 16.2 (0.2) | 25 (0.3) | 0.4 (0.1) | 3.1 (0.3) |
| | 6 | Pred-C | 23.4 (0.1) | 23 (0.1) | 3.4 (0.1) | 18.8 (0.1) | 29 (0.2) | 1 (0.1) | 5.8 (0.3) |
| | 6 | Pred-P | 24 (0.2) | 23.7 (0.2) | 3.7 (0.1) | 18.4 (0.2) | 30.5 (0.4) | 0.5 (0.1) | 3.3 (0.4) |
| | 7 | Pred-C | 26 (0.1) | 25.3 (0.1) | 4 (0.1) | 20.9 (0.1) | 33.2 (0.5) | 1.3 (0.1) | 5.9 (0.4) |
| | 7 | Pred-P | 27 (0.2) | 26.6 (0.2) | 4.4 (0.2) | 20.4 (0.3) | 34.7 (0.5) | 0.5 (0.1) | 3.4 (0.4) |
| | 8 | Pred-C | 30.3 (0.1) | 29.3 (0.2) | 5.2 (0.1) | 24 (0.1) | 40.3 (0.2) | 1.4 (0.1) | 5.9 (0.2) |
| | 8 | Pred-P | 30.3 (0.2) | 29.9 (0.3) | 5.2 (0.2) | 22.5 (0.4) | 39.5 (0.7) | 0.5 (0.1) | 3.5 (0.5) |
| | 9 | Pred-C | 33.9 (0.1) | 32.8 (0.1) | 6.2 (0.1) | 26.1 (0.1) | 44.7 (0.6) | 1.3 (0.1) | 5.8 (0.3) |
| | 9 | Pred-P | 34.2 (0.3) | 33.6 (0.4) | 6.4 (0.3) | 24.7 (0.5) | 45.5 (0.8) | 0.5 (0.2) | 3.6 (0.7) |
| | 10 | Pred-C | 39.6 (0.1) | 38.2 (0.2) | 8.6 (0.1) | 29.1 (0.1) | 57.8 (0.3) | 1.5 (0) | 6.3 (0.2) |
| | 10 | Pred-P | 38.5 (0.4) | 37.7 (0.5) | 8 (0.3) | 27 (0.6) | 52.7 (1.1) | 0.6 (0.2) | 3.6 (0.6) |
| | 11 | Pred-C | 44 (0.1) | 41.7 (0.1) | 10.1 (0.1) | 31.6 (0.1) | 63 (0.3) | 1.2 (0) | 4.9 (0.1) |
| | 11 | Pred-P | 43.3 (0.4) | 42.3 (0.5) | 9.8 (0.4) | 29.3 (0.6) | 60.8 (1.3) | 0.7 (0.2) | 3.8 (0.6) |
| BMI | 4 | Pred-C | 104.8 (0.1) | 104.6 (0.2) | 3.7 (0.1) | 99 (0.2) | 111.2 (0.5) | 0.3 (0.1) | 3.3 (0.3) |
| | 4 | Pred-P | 105.1 (0.2) | 105 (0.2) | 4 (0.1) | 98.7 (0.4) | 111.8 (0.5) | 0.1 (0.1) | 3.1 (0.3) |
| | 5 | Pred-C | 112.7 (0.1) | 112.6 (0.1) | 4.2 (0.1) | 106.2 (0.3) | 119.9 (0.4) | 0.4 (0.1) | 3.3 (0.1) |
| | 5 | Pred-P | 112.9 (0.2) | 112.8 (0.3) | 4.6 (0.2) | 105.6 (0.5) | 120.5 (0.4) | 0.1 (0.1) | 3 (0.2) |
| | 6 | Pred-C | 121.6 (0.1) | 121.1 (0.1) | 4.8 (0.1) | 114.2 (0.3) | 130 (0.2) | 0.3 (0.1) | 3.2 (0.1) |
| | 6 | Pred-P | 122.2 (0.2) | 122 (0.3) | 5.2 (0.1) | 113.8 (0.4) | 130.8 (0.5) | 0.1 (0.1) | 3 (0.2) |
| | 7 | Pred-C | 126.2 (0.1) | 125.7 (0.1) | 5.4 (0.1) | 118.2 (0.2) | 136 (0.4) | 0.4 (0.1) | 3.5 (0.1) |
| | 7 | Pred-P | 127.3 (0.3) | 127.2 (0.4) | 5.3 (0.2) | 118.8 (0.4) | 136.2 (0.5) | 0.1 (0.1) | 3 (0.2) |
| | 8 | Pred-C | 132.4 (0.1) | 132.2 (0.1) | 5.2 (0.1) | 124.1 (0.3) | 141.3 (0.2) | 0.1 (0.1) | 2.8 (0.1) |
| | 8 | Pred-P | 132.6 (0.2) | 132.4 (0.3) | 5.5 (0.2) | 123.8 (0.5) | 141.7 (0.6) | 0.1 (0.1) | 3 (0.3) |
| | 9 | Pred-C | 137.4 (0.1) | 137.1 (0.2) | 5.6 (0.1) | 128.1 (0.2) | 146.3 (0.4) | 0.1 (0.1) | 2.9 (0.1) |
| | 9 | Pred-P | 138.2 (0.3) | 138.1 (0.4) | 5.8 (0.2) | 128.9 (0.6) | 147.9 (0.6) | 0.1 (0.1) | 3 (0.3) |
| | 10 | Pred-C | 144.4 (0.1) | 144.3 (0.1) | 5.9 (0.1) | 134.9 (0.2) | 154.6 (0.2) | 0.2 (0) | 3 (0.1) |
| | 10 | Pred-P | 143.9 (0.3) | 143.8 (0.4) | 6.2 (0.2) | 134 (0.6) | 154.2 (0.7) | 0.1 (0.1) | 2.9 (0.2) |
| | 11 | Pred-C | 150.7 (0.1) | 150.6 (0.1) | 6.6 (0) | 140.2 (0.1) | 161.9 (0.1) | 0.2 (0) | 2.8 (0.1) |
| | 11 | Pred-P | 149.9 (0.3) | 149.7 (0.4) | 6.6 (0.2) | 139.2 (0.6) | 161 (0.7) | 0.1 (0.1) | 3 (0.2) |

## 5 | SUPPLEMENTARY MATERIAL E

Stan code for the bivariate HLM described in the study.

```
data {
   int<lower=0> Nobs;
   int<lower=0> Npreds;
   int<lower=0> Nresponsevals;
   int<lower=0> Netas_per_response;
   int<lower=0> Ngroups;
   matrix[Nobs,Nresponsevals] y_hw;
   vector[Nobs] agevar;
   int<lower=1> group[Nobs];
}
parameters {
   matrix[Npreds,Nresponsevals] beta0;
   matrix[Npreds,Nresponsevals] beta1;
   vector<lower=0>[Nresponsevals] sigmaeps; //sigmaeps_h, sigmaeps_w
   matrix[Ngroups,Netas_per_response*2] u;
sigma_matrix_prior;
 sigma_matrix;
   corr_matrix[Netas_per_response*2] Omega;        // prior correlation
   vector<lower=0>[Netas_per_response*2] tau;      // prior scale
}

transformed parameters {
matrix[Netas_per_response,Netas_per_response] sigma_matrix_epsilon;
matrix[Nobs,Nresponsevals] yhats;
matrix[Ngroups,Netas_per_response*2] eta; //eta0+eta1
sigma_matrix_epsilon = [[sigmaeps[1]^2, 0],[0, sigmaeps[2]^2]];

for(i in 1:Nobs){
eta[group[i],1:2]=to_row_vector(beta0)+u[group[i],1:2];
eta[group[i],3:4]=to_row_vector(beta1)+u[group[i],3:4];
yhats[i,]=to_row_vector(eta[group[i],1:2])+
to_row_vector(eta[group[i],3:4])*agevar[i];
}
}

model {
   //assign priors to beta0 and beta1
   to_vector(beta0[,1]) ~ normal(4,1);
   to_vector(beta0[,2]) ~ normal(2.6,1);
   to_vector(beta1[,1]) ~ normal(0.066,1);
   to_vector(beta1[,2]) ~ normal(0.130,1);

   tau ~ cauchy(0, 2.5);
   Omega ~ lkj_corr(Netas_per_response*2);
   sigmaeps ~ inv_gamma(0.5,0.5);


for(i in 1:Ngroups){
u[i,] ~ multi_normal([0,0,0,0],quad_form_diag(Omega, tau));
```

```
}

for (i in 1:Nobs) y_hw[i,] ~ multi_normal(yhats[i,], sigma_matrix_epsilon);
}

generated quantities {
  vector[Nobs] log_lik;
  matrix[Nobs,Nresponsevals] y_pred; // predcitions for observed values

  for (n in 1:Nobs){
      log_lik[n] = normal_lpdf(y_hw[n] | yhats[n], sigmaeps);
       y_pred[n,] = to_row_vector(multi_normal_rng(yhats[n,],
       sigma_matrix_epsilon));
  }
}
```

## 6 | SUPPLEMENTARY MATERIAL F

Figures S1, S2 and S3 illustate the evaluation results of the individual parameter-based (Pred-I) method versus population parameter-based prediction (Pred-P) method, with the aim of making predictions for the Contributing Group-2021 (defined in Section 2).

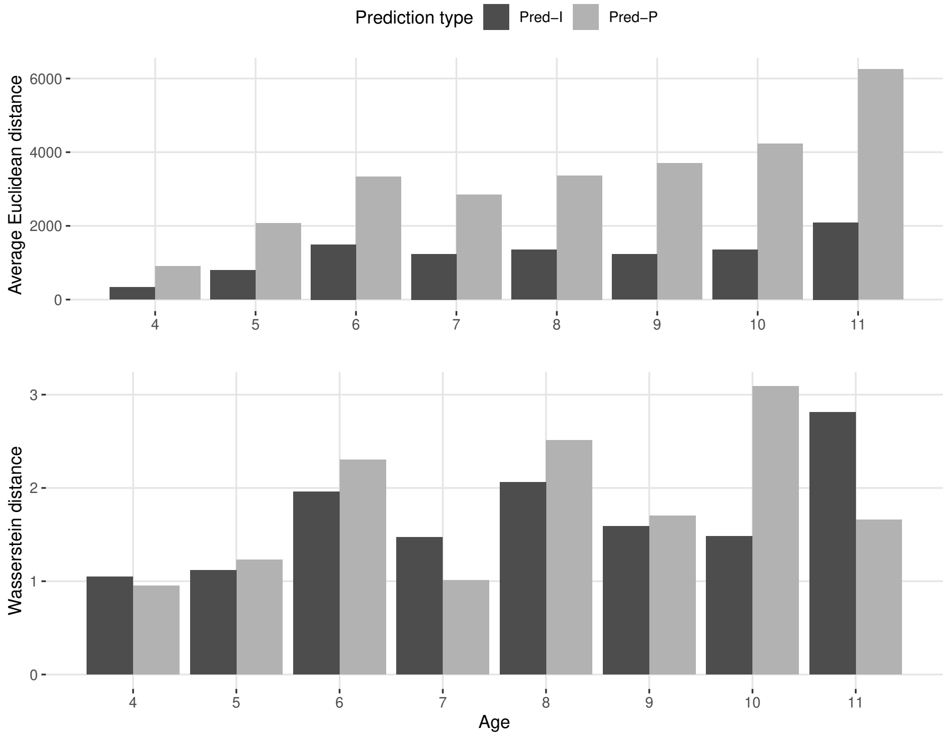


**FIGURE S1** Average Wasserstein and Euclidean distances for the Contributing Group-2021 (defined in Section 2) by age. Dark grey bars represent individual parameterbased predictions (Pred-I), and pale grey bars represent population parameterbased predictions (Pred-P). Pred-P and Pred-I indicate statistical distances between corresponding predictions and the Contributing Group-2021 observations.

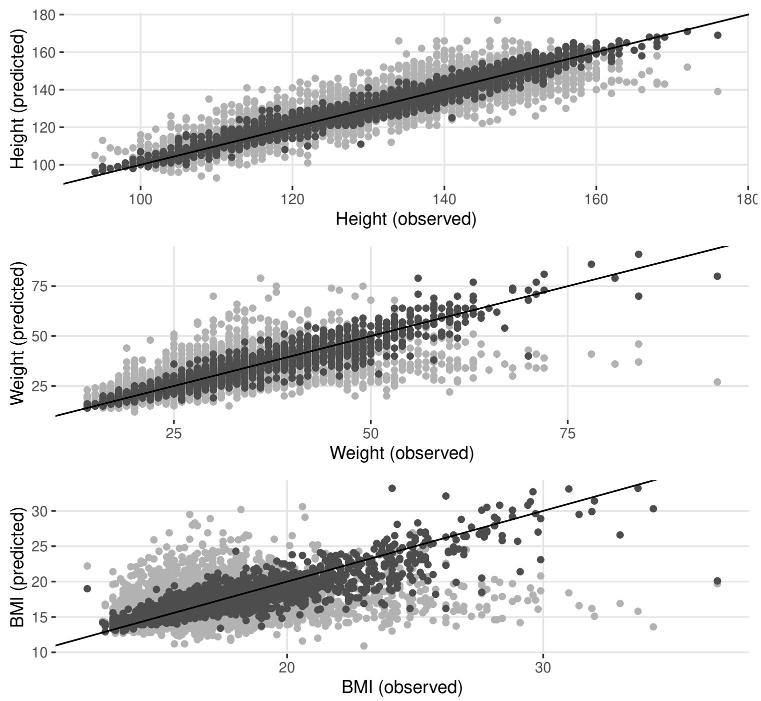


**FIGURE S2** All measurements of the Contributing Group-2021 (all children with individual parameters) plotted against predictions obtained by the Pred-P and Pred-I methods. The abbreviation Pred-P stands for population parameter-based prediction, and Pred-I stands for individual parameter-based prediction. Dark grey indicates Pred-I and pale grey Pred-P.

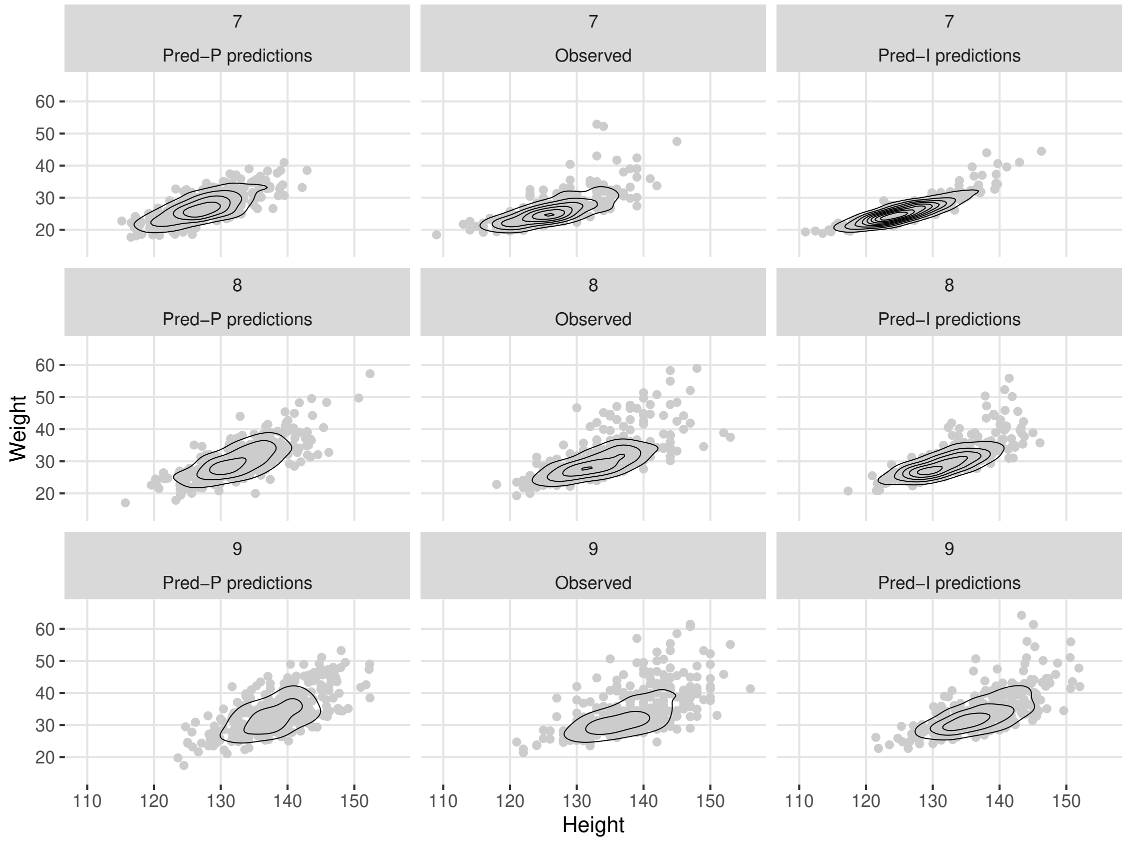


**FIGURE S3** Illustration for joint height and weight predictions obtained by the Pred-P and Pred-I methods for observations of the Contributing Group-2021 (ages 7-9) (defined in Section 2). The abbreviation Pred-P stands for population parameter-based prediction, and Pred-I stands for individual parameter-based prediction.

## 7 | SUPPLEMENTARY MATERIAL G

Table S5 describing the distribution of the Contributing Group's measurements (2014-2020) (defined in Section 2).

**TABLE S5** Statistics of the Contributing Group's measurements. For all ages, median and 5% and 95% quantiles are given.

| Age (years) | N | Median (Q05, Q95) | | | Skewness (kurtosis) | | |
|---|---|---|---|---|---|---|---|
| | | Height (*cm*) | Weight (*kg*) | BMI ($kg/m^2$) | Height (*cm*) | Weight (*kg*) | BMI ($kg/m^2$) |
| 2 | 2705 | 90 (84, 99) | 13 (11, 17) | 16 (15, 19) | 0.52 (3.06) | 0.6 (3.62) | 0.32 (3.15) |
| 3 | 3250 | 98 (92, 106) | 16 (13, 19) | 16 (14, 18) | 0.44 (3.17) | 0.83 (4.8) | 0.67 (4.86) |
| 4 | 3483 | 105 (98, 114) | 18 (15, 22) | 16 (14, 18) | 0.39 (3.35) | 1.21 (6.95) | 1.12 (6.88) |
| 5 | 3672 | 113 (105, 122) | 20 (16, 25) | 16 (14, 18) | 0.29 (3.23) | 1.65 (9.59) | 1.86 (10.37) |
| 6 | 4423 | 120 (112, 130) | 23 (19, 30) | 16 (14, 19) | 0.18 (3.11) | 1.37 (6.66) | 1.7 (8.57) |
| 7 | 3879 | 127 (118, 136) | 26 (21, 36) | 16 (14, 21) | 0.08 (3.08) | 1.95 (11.97) | 3.32 (35.47) |
| 8 | 3585 | 134 (123, 143) | 29 (23, 42) | 16 (14, 22) | 0.07 (3.02) | 1.72 (10.16) | 2.07 (13.62) |
| 9 | 3813 | 139 (129, 149) | 33 (26, 48) | 17 (14, 23) | 0.15 (3.12) | 1.49 (6.49) | 1.6 (6.82) |
| 10 | 3743 | 144 (134, 155) | 37 (28, 56) | 18 (15, 25) | 0.08 (3.12) | 1.4 (6.12) | 1.46 (5.84) |
| 11 | 3485 | 149 (138, 161) | 40 (30, 64) | 18 (15, 26) | 0.23 (3.19) | 1.42 (5.86) | 1.45 (5.71) |

## 8 | SUPPLEMENTARY MATERIAL H

Table S6 shows parameter estimates used in the Pred-C approach, where individual parameters are used when available and otherwise, population-based parameters are applied.

**TABLE** S6 Parameter estimates used in prediction (Pred-C approach). $M1$, $M2$ denote contribution to the Model 1 (2-5 year olds) and Model 2 (6-11 year olds), respectively. Subscripts 1 and 2 denote parameters estimated at level 1 and level 2. Level 1 parameters are used in Pred-I approach, and Level 2 parameters are used in Pred-P approach. Pred-C is a mixture of those approaches. $M1_1^*$ denotes the intercept term based on the prediction for individual $i$ at age 6.

| Age in 2021 | $\eta_{0i}$ | | | | $\eta_{1i}$ | | | |
|---|---|---|---|---|---|---|---|---|
| | 2-5 | 6-11 | 2-5 and 6-11 | neither | 2-5 | 6-11 | 2-5 and 6-11 | neither |
| 4 | $M1_1$ | $M1_2$ | $M1_1$ | $M1_2$ | $M1_1$ | $M1_2$ | $M1_1$ | $M1_2$ |
| 5 | $M1_1$ | $M1_2$ | $M1_1$ | $M1_2$ | $M1_1$ | $M1_2$ | $M1_1$ | $M1_2$ |
| 6 | $M1_1^*$ | $M2_2$ | $M2_1$ | $M2_2$ | $M2_2$ | $M2_1$ | $M2_1$ | $M2_2$ |
| ... | ... | ... | ... | ... | ... | ... | ... | ... |
| 11 | $M1_1^*$ | $M2_2$ | $M2_1$ | $M2_2$ | $M2_2$ | $M2_1$ | $M2_1$ | $M2_2$ |

**REFERENCES**

1. Feng C, Wang H, Lu N, Tu X. Log transformation: application and interpretation in biomedical research. *Statistics in medicine.* 2013;32(2):230–239.
2. Kauppala T. Developmental trajectories of height, weight and BMI across childhood: Bayesian hierarchical modeling of longitudinal data. *University of Helsinki, Faculty of Science.* 2021.
